# Bioethics: a look at animal testing in medicine and cosmetics in the UK

**DOI:** 10.18502/jmehm.v12i15.1875

**Published:** 2019-11-12

**Authors:** Stefane Kabene, Said Baadel

**Affiliations:** *Faculty of Communication, Arts and Sciences (FCAS), Canadian University Dubai, Dubai, UAE.*

**Keywords:** Animal testing, Bioethics, Cosmetics testing, Medical research

## Abstract

Using animals for cosmetics and medical tests has contributed towards a debate based on conflicting interests. Despite the efforts in justifying the value of animals in conducting analyses, this study seeks to elaborate whether or not it is rational to use animals as test subjects in medical and cosmetics fields. The value of animal life is at the core of the emotional conflicts that arise when animals become experimental subjects in medical and cosmetics fields. The aim of this study is to determine if there are ethical differences in the use of animal testing in medicine versus cosmetics. The research, through review and content analysis of the existing literature, compares and provides the outcomes of using animals in medical and cosmetics tests by examining studies conducted in the UK. The findings of this research indicated that animal testing is considered acceptable in the medical field only if there are no other alternatives, but is completely unacceptable in the cosmetics field. The study also provides recommendations in the form of alternatives that protect animals from cruelty and may benefit the different stakeholders and the society at large.

## Introduction

Throughout history, animals have been the subject of experimentation to improve our understanding of anatomy and pathology (1). However, animal testing only became significant in the twentieth century (2). 

Animal experiments are used extensively when developing new medicines and for testing the safety of certain products. Recently, the use of animals for biomedical research has been severely criticized by animal rights and protection groups. Similarly, many nations have established laws to make the practice of animal testing more humane. There are two positions in animal testing. One is that animal testing is acceptable if suffering is minimized and there are human benefits that could not have been achieved using any other means (3). The second position considers animal testing unacceptable because it causes suffering, and the benefits to human beings are either not proven or could be obtained using other methods.

As such, animal testing is a highly controversial subject that often elicits conflicting emotions from supporters and critics alike. It is also a divisive subject as some people support animal testing only in certain cases and oppose its use in other areas. For example, scientists note that significant medical breakthroughs have only been made possible through drug testing on animals. To them and other like-minded people, such achievements are reason enough to keep using animals in the lab (4). Animal tests determine if experimental drugs are effective or ineffective on human beings. Eventually, the medicine is tried out on a small group of humans through clinical trials before declaring the medicine safe to use. 

Badyal and DesaI (5) note that these treatments are as beneficial to humans as they are to animals, since some human diseases are found in animals too. Therefore, some who support animal testing only advocate its use for medical (but not cosmetics) purposes, arguing that the advancement in human medicine may lead to advancement in animal medicine. 

While a significant population completely disapproves of animal testing, a faction of people only disagrees with the use of animals for cosmetics testing, arguing that it is despicable and cruel to use animal life merely so that humans can advance their beauty technology. The concern extends to animals used for science, and people want animal suffering to be minimized (6). The discovery of new drugs has for a long time been based on a number of interactions among aspects such as data collected from patients, tissues, organs or cell culture and varied animal species (7). Those who oppose the use of animal testing for cosmetics believe it is outrageous and cruel to use animal life for the simple reason of making humans look better, and that the benefits to human beings do not validate the harms done to animals (7).

For such reasons, the use of animals for testing cosmetics products has been banned in the UK and all other member states of the European Union since 2013 (8). However, other countries like China and the United States of America still continue with the practice (9). Linzey adds that about 50 - 100 million animals are used for experiments every year, and that over 1.37 million animals were used for drug experimentation in America in the year 2010 (9). In the meantime, the number of experiments conducted on animals has declined in Britain but is increasing in other countries. While experiments involving vertebrates are regulated in most countries, experiments on invertebrates are not (5). 

The aim of this study is to examine whether or not animal testing is still useful and necessary in the present time, and whether there are ethical differences between animal testing in medical and cosmetics fields. We use the UK as our case study and provide alternatives that can be recommended in place of animal testing.

## Methods

This review was based on a cross-sectional survey by Clemence and Leaman (11) that analysed the importance of animal testing from two different aspects: medicine and cosmetics. The population consisted of individuals residing in the UK, and the sample size was 987 (= 0.03). The research included 496 men and 491 women. The report compared public views with the responses from a similar study in 2014 that had 969 participants (477 men and 492 women). The inclusion criteria were based on numerous strata such as gender, social grade definitions (i.e., professionals such as doctors and architects, people with responsible jobs such as professors, middle rank public servants such as nurses and clerics, skilled manual workers, etc.), respondents’ working status (fulltime, part-time, not working), ethnicity (white, non-white), and educational background. This report measured public perception on whether it is ethical to use animal testing for medical or cosmetics purposes. Participants were required to state whether they found it acceptable, mostly unacceptable, unacceptable, or were undecided. Consequently, the same participants were also tasked to indicate whether they saw conducting animal testing for scientific experimentation as completely necessary, somewhat necessary, not very necessary, completely unnecessary, or they did not know.

The study also utilized data from the UK Home Office (12) to determine which animals were most frequently used for medical and cosmetics research around the world. This report also provided crucial information as to the purposes of animal testing, for instance for medical research, biological testing, regulatory testing, etc.

## Results

According to the UK Home Office (12), in the year 2016, 48.6% of the animal tests in medical research were conducted for genetically oriented studies. Moreover, 28.5% of the medical research involving animal testing was for basic biological research, 13.5% was for regulatory

**Fig. 1:**
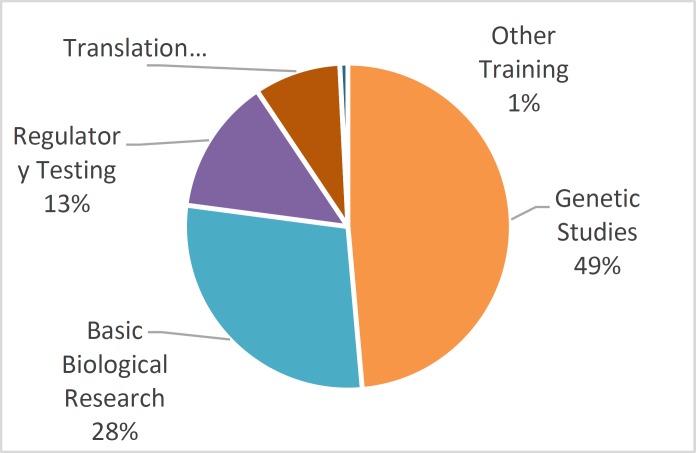
Purposes of Animal Testing in Medicine

testing, 8.6% was for translating research from animals to humans, and 0.8% for other trainings. This is summarized in Figure 1 below. 

**Fig. 2:**
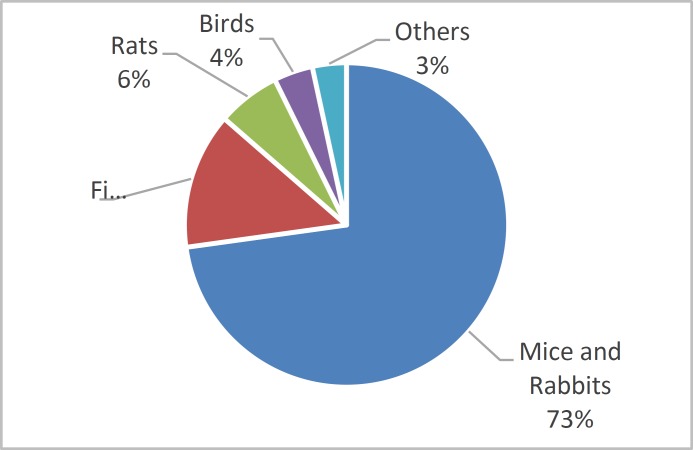
Types of Animals Used in Testing

Data from the UK Home Office (10) indicates that the most commonly used animals for medical and cosmetics research are mice and rabbits (72.8%), fish (13.6%), rats (6.3%), birds (3.9%) and other animal species representing 3.4% of the total test animal population, as indicated in Figure 2 below. 

**Fig. 3:**
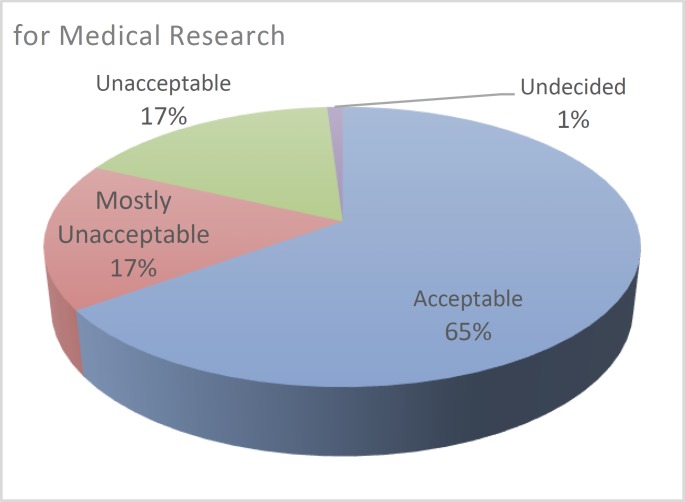
Animal Testing for Medical Research

**Fig. 4:**
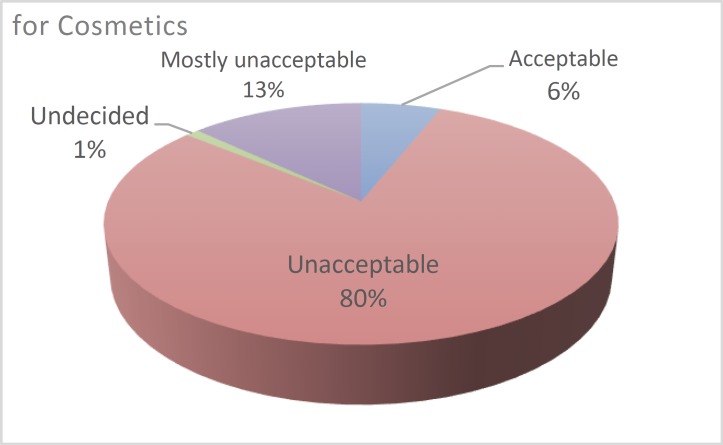
Animal Testing for Cosmetics Research

A published report (12) indicated that 17% of the sampled group viewed animal testing for medical research as ‘mostly unacceptable’ if there were no alternative, 17% as ‘not acceptable’, and 65% as ‘acceptable’. This was in stark contrast with testing for cosmetics purposes, to which an overwhelming 80% of the participants responded as ‘unacceptable’. The summary of the results is provided in Figure 3 and Figure 4 below.

**Fig. 5:**
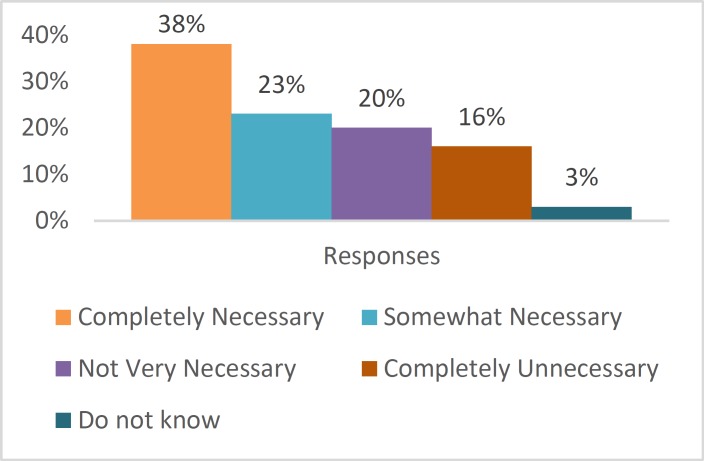
Necessity of Conducting Scientific Experiments on Animals

 In the same study (12), the participants were asked about the necessity of conducting scientific experiments on animals, which 38% of the respondents viewed as ‘completely necessary’, 23% as ‘somewhat necessary’, 20% as ‘not very necessary’, and 16% as ‘completely unnecessary’. The results are summarized in Figure 5 below.

The application of these methods to evaluate the safety of cosmetics was the most detested as stated by about 80% of the people who were interviewed during the investigation. The sensitivity to human life, on the other hand, reduces the strictness towards utilization of animals to find anti-viruses and antibiotics for various diseases.

## Discussion

The outcome portrays the essentiality of using animals to determine materials that would help the population to live healthily (13). However, in the past few decades, the number of animals used for testing drugs has been steadily decreasing (14).

The data indicates that most of the medical research processes involving animal testing emanate from genetically oriented studies, which constitute 48.6% of the medical research animal testing. Experimentation on human genetics presents various legal and ethical challenges to medical and biological researchers, alongside problems in creating experimental procedures using human test subjects. These problems occur partially due to the fact that the experimentation processes involved in these types of studies often lead to extensive gene and physiological damages to the test subjects. Such experiments typically involve deliberate presentation of diseases and other gene modifications to the test subjects, usually requiring the euthanizing of the involved subjects (15). The animal testing experimentations involving genetic processes include studies in gene modification and examine diseases believed to hold genetic components, such as cancer and diabetes (16). These experimentation processes typically involve some sort of gene modification that can simulate the presentation of genetically based disorders manifested in human beings to allow researchers to better understand those disorders.

The data also indicate that another major application of animal testing in the medical field is in basic research in biological systems and processes, which accounts for 28.5% of the testing categories. This application of animal testing in medical research involves studies in how biological systems function, and the nature and manner of disease transmission in living organisms. The findings accrued through these kinds of studies translate to advancements in the scientific knowledge of human pathology and present opportunities for the derivation and testing of cures, as noted by Festing and Wilkinson (17). 

The findings further present that regulatory testing (13.5%) and animal to human translation research (8.6%) account for significant portions of the application of animal testing in the medical field. The use of animal testing for regulatory testing purposes involves applying new medical findings, procedures and products to animals to see if they meet the thresholds mandated by the medical regulatory bodies. Translation of research findings from animals to humans involves conducting research into the possibility of animal pathogens becoming infectious to humans, and identifying potential ways of applying non-human physiology to the improvement of human health. Other forms of medical and biological trainings and studies that also engage the use of animals in experimentation in the medical field include elements such as basic physiology and pathogen studies, typically conducted in educational institutions.

Animal testing in the field of cosmetics generally involves the use of animal subjects in testing new cosmetics products and ingredients. The practice essentially involves the application or forced ingestion or injection of these substances to various parts of test animals to examine their toxicity, irritation of the eyes and/or skin, ultraviolet light-triggered toxicity, and their potential for causing unwanted gene mutations (18). 

The use of animal testing in the field of cosmetics research and production presents an unethical viewpoint since the findings do not advance human health, and the practice leads to the torture and killing of animals. The Humane Society (18) also notes that at the conclusion of the experimentation, the animals are usually killed through methods such as decapitation, neck twisting and asphyxiation, often without pain relief. 

With regard to the ethical principles of animal testing in both fields, a convincing argument should first be presented to the Institutional Animal Care and Use Committee (IACUC). This is to justify the need for a researcher to conduct animal studies, and to ensure that the research is conducted using the smallest possible number of animals and with minimal suffering. Additionally, Naderi et al. (19) noted an increased level of legislation on the matter of animal testing, with researchers being required to submit comprehensive proposals to the IACUC to demonstrate procedural compliance with the guiding principles of the organization before conducting animal tests. Furthermore, Holden (20) highlighted the fact that researchers need to justify to review and ethics committees the use of mice rather than other alternatives in experiments. These issues indicate that researchers should look for alternatives to animal testing before proceeding with animal trials.

The issue then remains on the nature and availability of alternatives to animal testing in the medical research field. Researchers have undertaken measures to introduce some levels of such alternatives in medical studies. The accrued data indicate that a significant number of people agree with animal testing for medical research, especially when compared to those who agree with animal testing for cosmetics purposes. The data obtained from the studies indicate a slow but perceptible shift in the public opinions regarding animal testing for medical research purposes. People are increasingly finding it unacceptable to use animal test subjects even in medical research. However, the majority of the sampled people believed that medical testing procedures should use animal test subjects, but only when there is no other alternative. This indicates that people view animal testing for medical research as ethical, but under certain conditions.

The use of animals in research is still relevant because the process is useful in veterinary medicine as it helps the students understand the physiology and anatomy and improves surgical skills (21). The study by Badyal and Desai (5) supports this perception by highlighting the fact that animal use in laboratory investigation will make new discoveries possible. However, researchers should apply ethical concepts to reduce the amount of pain and unnecessary procedures for the animals. Moreover, animal testing to develop new drugs will continue to protect the future existence of humanity. Cheluvappa, et al. (22) reiterate that animal experimentation will remain essential to testing future medicine because it helps scientists understand the changes of behaviour, embryology and genetics through dissections that are conducted on the genetically produced animals.

Animals play an important role in testing human drugs as they have a large number of medical reactions similar to those of human beings. Specifically, animals such as dogs, mice and rabbits have an identical DNA that cannot be replicated through artificial models. Public concern for the increasing use of animals in terms of ethics and safety provokes anxiety among the population. Conversely, these uncertainties and unavailability of trustable alternatives show the importance of using animals in medical research as the scientists aim to protect the human race (23). 

However, the use of animals to test cosmetics is highly limited due to the availability of alternative sources. For instance, The Laboratory Animals Veterinary Association (LAVA) claims that the UK government prohibits any individual from using animals to determine the suitability of cosmetics to the human body (13, 24). In its circular, The European Union states that they have succeeded in developing alternative measures that cosmetics firms can apply to test their products without using laboratory animals (25).

**Recommendations: Alternatives to Animal Testing**

To improve business ethics in cosmetics companies, it is necessary for alternatives to be integrated instead of animals. Companies can employ assessment of scientific barriers to find replacements for animal test subjects and to procure the knowledge of correctly using animals for medical and cosmetics tests. Sophisticated tests on human cells or tissues, computer-modelling techniques, and experiments on people who volunteer are some measures that can limit acts of animal cruelty by cosmetics companies. Companies need to integrate tests that minimize involvement of animals in order to limit the possibility of animal cruelty, and consequently improve their business ethics. Some of the recommended alternatives are listed here.

***Computer Simulation***

The concept was developed by Denis Noble, and the system is currently enrolled in clinical settings. These simulations are used to test heart replacements, and are also applied to explore human behavior. Various scholars provide that this model is more accurate than animal experiments because it uses human data to analyse diseases and make predictions (26). 

***Stem Cells***

Stem cells are proper alternatives to the in vitro systems of disease testing and toxin evaluations (27). The experiments involve evaluation of embryonic stem cells that can be grown in Petri dishes. The Petri dishes can be placed in the cells, and after that the resulting components are placed under evaluation to help in the discovery of new medications. Stem cells are essential because they can differentiate into human tissues and make it possible to screen the suspected diseases (26). 

***Biochips***

These materials are majorly utilized in the cosmetics industry to minimize the number of animals used to test the level of toxicity in a product. Significantly, investigations showed that human tissues developed in laboratories can be used to assess the allergic responses to the available chemicals (28). These results can then be analysed by comparing reactions, and a bio signature of genes is used to make appropriate interventions. 

***3D Images***

Notably, scientists can take high-resolution pictures of human tissues, which are then analyzed with the help of various computer systems. The advantage of this model is characterized by its ability to customize the parts of the organism under consideration. Moreover, 3D images also develop prototype designs and materials that can be used to investigate the existing and future ailments (29).

## Conclusion

This study indicates that it is justifiable to use animals in experimentations only when there are no alternatives, and the tests have significant benefits to humans. Many researchers are working towards finding options that will help eliminate the use of animals for medical and cosmetics tests. The different natures of tests conducted on animals in the fields of medicine and cosmetics tend to have clear negative implications. For such reasons, it is imperative for organizations to develop practices that endorse business ethics. Although animal tests are ideal in establishing whether drugs can be effective in treating humans for various ailments, entities that conduct these tests need to be educated about the gravity of the situation. Animals have been extremely useful in conducting genetic studies and for biological systems investigations. However, a comparison between animal tests in medicine and cosmetics reveals that their benefits in the field of medicine outweigh those in cosmetics. Therefore, animals are essential contributors to scientific experiments that are affiliated with the medical industry. The effects that medical products may have on humans make it ethical to carry out the tests on animals first. 

After analysing the arguments of both the supporters and opponents involved in the controversial subject of animal testing, it is difficult to determine which direction is right or wrong. However, the agreement is that animal suffering be minimized at all costs. This research concludes that cosmetics companies should adhere to the established laws and principles against the use and abuse of animals in tests and should seek alternative methods to test their products. 

## Notes:

**Citation to this article:**

Kabene S, Baadel S. Bioethics: a look at animal testing in medicine and cosmetics in the UK. J Med Ethics Hist Med. 2019; 12: 15.
